# The Calcium-Sensing Receptor is A Marker and Potential Driver of Neuroendocrine Differentiation in Prostate Cancer

**DOI:** 10.3390/cancers12040860

**Published:** 2020-04-02

**Authors:** Fanny Bery, Mathilde Cancel, Aurélie Chantôme, Roseline Guibon, Franck Bruyère, François Rozet, Karine Mahéo, Gaëlle Fromont

**Affiliations:** 1 Inserm N2C UMR1069 “Nutrition, Croissance et Cancer” Université de Tours, CEDEX 1, F-37032 Tours, France; fanny.bery@etu.univ-tours.fr (F.B.); mathilde.cancel@univ-tours.fr (M.C.); aurelie.chantome@med.univ-tours.fr (A.C.); R.GUIBON@chu-tours.fr (R.G.); karine.maheo@univ-tours.fr (K.M.); 2 Department of Oncology, CHRU Bretonneau, CEDEX 9, F-37044 Tours, France; 3 Department of Pathology CHRU Bretonneau, CEDEX 9, F-37044 Tours, France; 4 Department of Urology, CHRU Bretonneau, CEDEX 9, F-37044 Tours, France; f.bruyere@chu-tours.fr; 5 Institut Mutualiste Montsouris, Department of Urology, F-75014 Paris, France; francois.rozet@imm.fr

**Keywords:** prostate cancer, calcium-sensing receptor, calcium, neuroendocrine differentiation, hormone therapy

## Abstract

The mechanisms underlying neuroendocrine (NE) differentiation in prostate cancer (PCa) remain mostly uncharacterized. Since a deregulated calcium homeostasis has been reported in neuroendocrine prostate cancer (NEPC), we explored herein the link between NE differentiation and the calcium-sensing receptor (CaSR). CaSR expression was evaluated by immunohistochemistry—together with NE markers—on tissue microarrays containing samples of normal prostate, localized PCa, metastatic castration resistant PCa (MCRPC) and NEPC. In prostate tissues, we observed a strong association between CaSR and chromogranin expression. Both markers were strongly expressed in all cases of NEPC and co-expression was confirmed by double immunostaining. In MCRPC, the expression of CaSR was significantly associated with shorter overall survival. The involvement of CaSR in NE differentiation was evaluated in PCa cell lines. Inhibition of CaSR led to decrease the expression of neuronal (NSE, βtubulinIII) and NE (chromogranin, synaptophysin) markers in the NE PCa cell line NCI-H660. A decrease of neuronal and NE markers was also observed in siCaSR-transfected PC3 and 22RV1 cells, respectively, whereas CaSR activation increased both NSE and synaptophysin expression in PC3 cells. These results strongly suggest that CaSR is a marker and a driver of NE differentiation in PCa and emphasize the potential of CaSR directed therapy for NEPC patients.

## 1. Introduction

Prostate cancer (PCa) is the most frequently diagnosed cancer among men and a common cause of male cancer death. Calcium (Ca^2+^) signaling has been involved in both the development and progression of PCa. Dietary calcium intake is associated with an increased risk of PCa and aggressive disease in several epidemiological studies [1,2,3,4]. This is most likely linked to the fact that changes in cytosolic Ca^2+^ and Ca^2+^ signal trigger key events in cancer cells, such as proliferation, invasion and resistance to apoptosis [5]. Disturbances of Ca^2+^ homeostasis can be caused by various mechanisms, such as excessive calcium intake in the diet, vitamin D deficiency, structural and functional changes in vitamin D receptor, changes in Ca^2+^ channels and Calcium-Sensing Receptor (CaSR) [6].

The CaSR is a G protein-coupled cell surface receptor (GCPR), characterized by a large extracellular domain that can bind several ligands, including Ca^2+^, amino acids, vitamin D and interleukin 6 [7]. The best documented role for CaSR is the systemic homeostasis of free ionized Ca^2+^ concentration, through the regulation of parathormone secretion by the parathyroid gland. CaSR is also involved in Ca^2+^ homeostasis in non calcitropic tissues, including brain, lung and breast, where it regulates several cellular processes, such as proliferation, differentiation and hormone secretion [7]. In PCa cell lines, CaSR was reported to be a mediator of Ca^2+^ effects on cell proliferation and tumor progression [8].

Neuroendocrine (NE) cells are normal and rare components of the human prostate, randomly scattered within the epithelium of the prostate glands. These cells contain a variety of neuropeptide and hormones, such as chromogranin A, synaptophysin, neuron-specific enolase (NSE) and others [9]. NE cells are defined in current practice by immunohistochemical positivity for either chromogranin, synaptophysin or CD56 [9]. NE cells can also be found after immunohistochemical staining in usual prostate adenocarcinoma, scattered among cancer cells. The presence of such a focal NE differentiation in hormone naive PCa has not been shown to worsen disease prognosis [9]. In contrast, neuroendocrine prostate cancer (NEPC) is a highly aggressive form of PCa, that rarely arises *de novo* and most often emerges from prostate adenocarcinoma after androgen deprivation or androgen receptor (AR) targeted therapy [10]. Although some NEPC are composed only by NE cells, characterized by the presence of secretory granules, others are admixed with an adenocarcinoma component [10]. NE differentiation of PCa cells has been associated with significant modifications of Ca^2+^ homeostasis compared to adenocarcinoma cells, with increased Ca^2+^ current density and overexpression of voltage-gated Ca^2+^ channel CaV3.2 [11]. This channel is involved in Ca^2+^ dependent secretion of neuroendocrine peptides [12]. The CaSR has been reported to be expressed in cells of the diffuse neuroendocrine system, including the endocrine pancreas [13] and the enteroendocrine cells of the gastrointestinal tract [14], where it regulates hormone secretion. CaSR is also expressed in lung epithelial cells, with a selective staining in NE cells [15].

We therefore hypothesized that NE differentiation in the normal and tumoral prostate could be linked to CaSR expression. The present study aimed to analyze CaSR expression at different stages of PCa progression including NEPC, in correlation with the neuroendocrine phenotype. In addition, we investigated in vitro the impact of CaSR modulation on NE differentiation in PCa cell lines.

## 2. Results

### 2.1. Prevalence of CaSR Expression and Correlation with Chromogranin Staining

The characteristics of immunostainings in the different groups of prostate tissues are summarized in Table 1.

**Figure 1:**
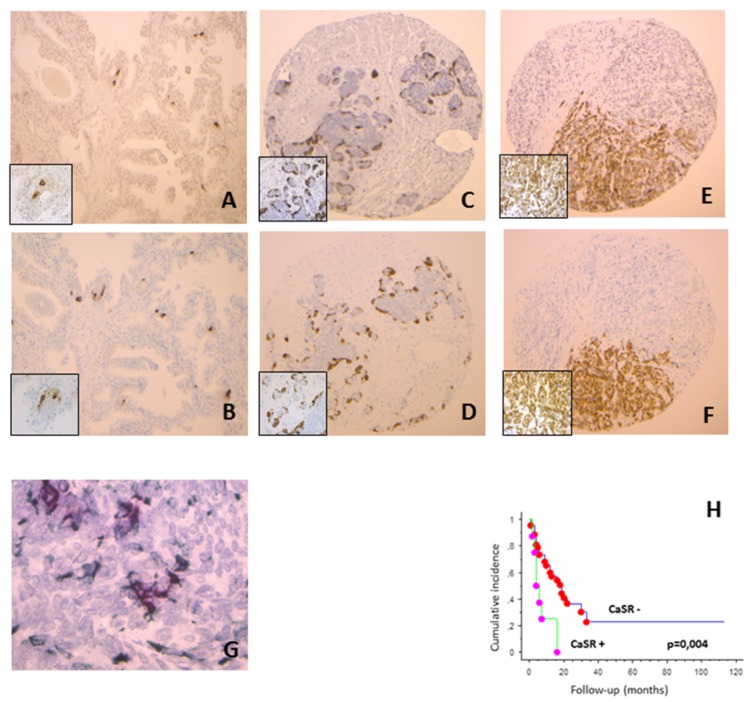
Immunostaining for calcium-sensing receptor (CaSR) (**A**,**C**,**E**) and chromogranin (**B**,**D**,**F**) on sequential slides. Both markers are expressed in the same areas of normal prostate tissue (**A**,**B**, ×10; in inset ×40) and metastatic castration resistant prostate cancerv (MCRPC) (**C**,**D**, ×5; in inset ×40). In case of mixed neuroendocrine prostate cancer (NEPC), only the neuroendocrine (NE) component expressed both CaSR and chromogranin (**E**,**F**, ×5; in inset ×40). (**G**). Double immunostaining showing co-localization of CaSR and chromogranin in MCRPC. Cytoplasmic expression of chromogranin is shown as red; membranous staining of CaSR is shown as green. In some cells, CaSR was expressed without chromogranin staining (×60). (**H**). Kaplan-Meier overall survival in MCRPC patients, according to CaSR staining (*p* = 0.004).

In normal prostate tissues, CaSR and chromogranin were expressed in the epithelial compartment in respectively 24 and 23 cases on 57, with less than 1% of positive cells (Figure 1A,B). CaSR staining was observed on cancer cells in 26% of CLC cases, 25% of MCRPC samples (Figure 1C) and 100% of NEPC (Figure 1E). Considering the percentage of positive cells per case, we observed an increase in CaSR staining between CLC, MCRPC and NEPC (Kruskall–Wallis, *p* < 0.0001). Similar findings were found for chromogranin expression (Kruskall–Wallis, *p* < 0.0001) (Figure 1D,F).

In normal prostate tissues, CLC and MCRPC, we observed a strong association between CaSR and chromogranin expression (Spearman test, *p* < 0.0001). In NEPC, both markers were expressed in each case. No significant difference was found in CaSR and chromogranin expression between de novo NEPC and treatment induced NEPC (Mann–Whitney, *p* = 0.17 and 0.09, respectively).

In NEPC, synaptophysin positivity was found only in 6 cases on 15, most often those with low chromogranin staining. In normal prostate tissues, CLC and MCRPC, synaptophysin staining was observed in approximately half of cases positive for chromogranin, and none of negative cases. Double immunostaining confirmed the co-expression of chromogranin and CaSR in all groups of tissues, including MCRPC and NEPC. In some cells, CaSR was expressed without chromogranin staining (Figure 1G).

### 2.2. Correlation of CaSR Expression with Proliferation and ERG Status. Association with ISUP Group and pTNM Stage in CLC

In the CLC and NEPC groups, no association was found between CaSR and proliferation, determined by the percentage of Ki67 positive cancer cells (Spearman test, *p* = 0.4 and 0.5, respectively) and between CaSR and the ERG status (Mann–Whitney, *p* = 0.1 and 0.9, respectively). In MCRPC, an inverse correlation was observed in one hand between CaSR and proliferation (Spearman test, *p* = 0.002), in the other hand between CaSR and ERG status (Mann–Whitney, *p* = 0.007). A similar inverse correlation was found between chromogranin and ERG status (Mann–Whitney, *p* = 0.001).

In the CLC group, no significant association was found between CaSR and the ISUP score (Kruskall–Wallis, *p* = 0.4). CaSR expression was increased in pT3 tumors compared to pT2 (Mann–Whitney, *p* = 0.02).

### 2.3. Prognostic Value of CaSR Staining

In CLC treated by radical prostatectomy, CaSR and chromogranin expressions were not significantly associated with biochemical relapse (log rank, *p* = 0.18 and 0.40, respectively); only Ki67 staining was significantly associated with biochemical progression, after adjusting for preoperative PSA, ISUP group and pTNM stage (log rank, *p* = 0.007). In MCRPC, CaSR expression was significantly associated with shorter overall survival (log rank, *p* = 0.004) (Figure 1H), while proliferation and chromogranin staining were not (log rank, *p* = 0.3 and 0.1, respectively).

### 2.4. Neuroendocrine Profile of PCa Cell Lines and CaSR Expression

**Figure 2:**
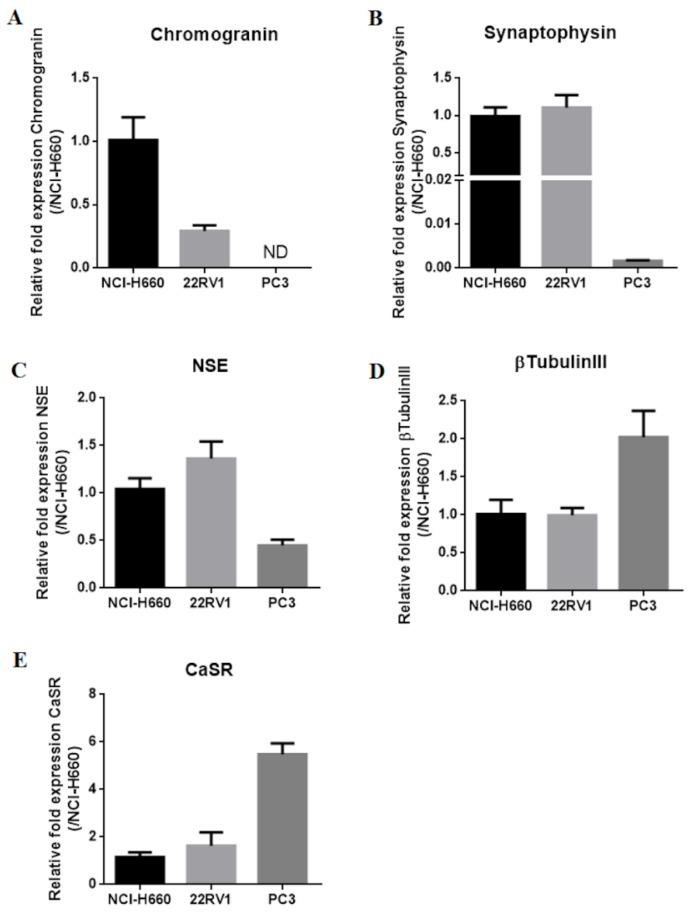
Neuroendocrine markers (**A**–**D**) in NCI-H660, PC3 and 22RV1 cell lines and Expression of CaSR (**E**). qPCR results (mean ± SEM) are expressed in 2^-ΔΔCt^ and normalized to NCI-H660 cell line.

**Figure 3:**
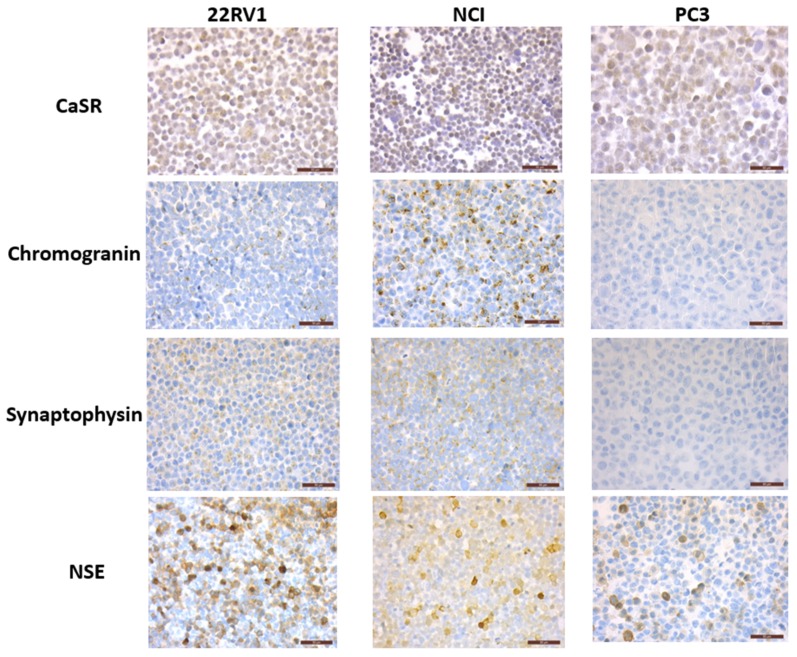
Expression of CaSR and neuroendocrine markers in NCI-H660, PC3 and 22RV1 cell lines. Immunohistochemical staining on cell pellets: all markers are expressed in NCI-H660 and 22RV1 cell lines, with nevertheless a weak expression of chromogranin in 22RV1. In PC3 cells, while both CaSR and NSE are expressed, no staining was observed for chromogranin and synaptophysin.

The expression of CaSR, neuronal markers (NSE and βtubulinIII) and NE markers (chromogranin and synaptophysin) was analyzed by qPCR and immunohistochemistry in the PCa cell lines NCI-H660, PC3 and 22RV1 (Figure 2 and Figure 3).

NCI-H660, the only existing cell line derived from human NEPC, was used as reference. As expected, in the NCI-H660 neuroendocrine cell line, all markers were highly expressed especially the neuroendocrine markers chromogranin and synaptophysin. Unfortunately, this cell line grows extremely slowly, with floating cell clusters that limit the use of the model.

The 22RV1 cell line shows a neuroendocrine expression profile similar to NCI-H660 (except for a lower chromogranin level), suggesting the acquisition of NE characteristics (Figure 2 and Figure 3).

In PC3 cells, chromogranin and synaptophysin were weakly expressed or undetectable. In contrast, consistent expression of neuronal markers was noticed, with a high βTubulinIII mRNA level and a sustained mRNA and protein level expression for NSE (Figure 2 and Figure 3). CaSR was expressed in the 3 PCa cell lines and with the highest mRNA level in PC3 (Figure 2 and Figure 3).

### 2.5. Pharmacological CaSR Regulators Induce Changes in NE Marker Expression

To evaluate the potential implication of CaSR in neuroendocrine differentiation, we treated the NE cell type NCI-H660 and 22RV1 with the CaSR inhibitor *NPS-2143,* and the neuronal cell type PC3 with R-568, a CaSR-positive allosteric modulator.

**Figure 4:**
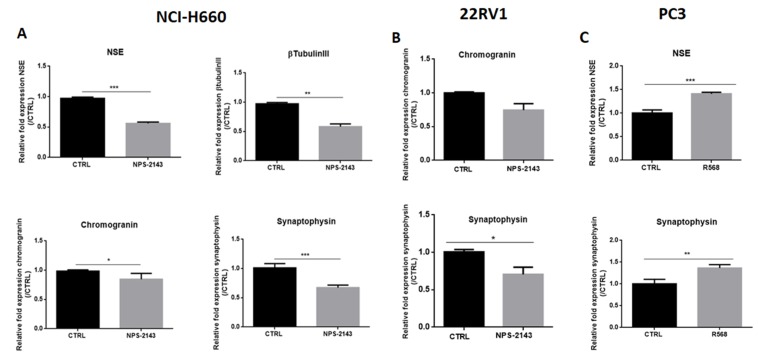
(**A**,**B**): Effects of CaSR inhibitor on the expression of NE markers in NCI-H660 and 22RV1. Cells were treated for 72 h (NCI-H660) or 24 h (22RV1) with a CaSR inhibitor, NPS-2143 at 1 µM (NCI-H660) or 300 nM (22RV1). (**C**): Effects of CaSR activator on the expression of NE markers in PC3. Cells were treated for 24 h with a CaSR activator, R-568 (10 µM) in presence of calcium (1.2 mM). qPCR results are normalized to control condition and are expressed as mean ± S.E.M. The statistical differences are indicated: *p < 0.05; **p < 0.01; ***p < 0.001 (Mann–Whitney). *n* = 3.

Decreased expression of NE markers was observed in NCI-H660 cells treated with *NPS-2143* compared to control cells. Indeed, in treated NCI-H660 cells, the expression of neuronal markers NSE (Figure 4A) and βtubulinIII (Figure 4) was reduced by 42% and 40% respectively and the expression of NE markers chromogranin (Figure 4A) and synaptophysin (Figure 4A) was reduced by 14% and 33%, respectively. In 22RV1 cells treated with NPS-2143, our results showed a decreased expression of synaptophysin by 30% and a decreasing tendency for chromogranin (Figure 4B). After treatment of PC3 cells with the CaSR activator R-568, NSE and synaptophysin mRNA levels were increased by 41% and 36% respectively (Figure 4C).

### 2.6. Downregulation of CaSR Reduces the Expression of Neuronal Markers in PC3 and Neuroendocrine Markers in 22RV1

**Figure 5:**
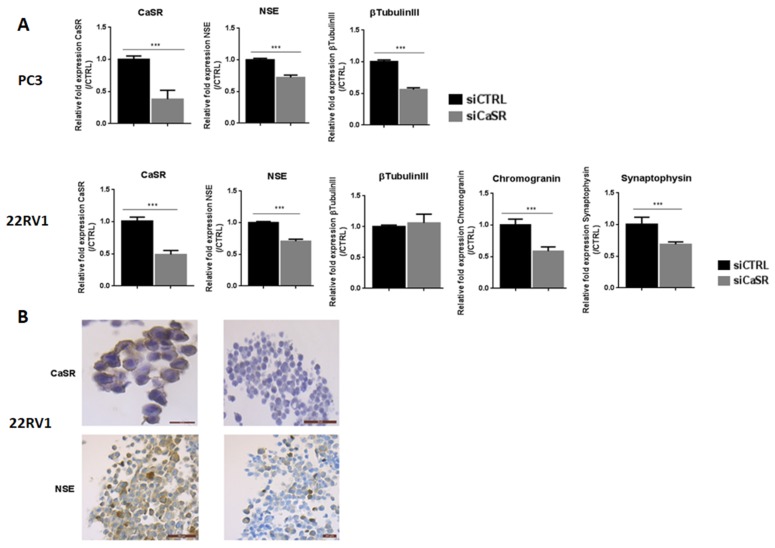
(**A**). Effects of CaSR inhibition on the expression of NE markers in PC3 and 22RV1. Cells were transfected with a siRNA control (siCTRL) or directed against CaSR (siCaSR) for 48 h (qPCR analysis were performed 48 h post-transfection). Results are normalized to control condition and are expressed as mean ± S.E.M. The statistical differences are indicated: ***p < 0.001 (Mann–Whitney). *n* = 3. (**B**). Immunohistochemical expression of CaSR and the neuronal marker NSE in 22RV1 cells, transfected with either a siRNA control (siCRTL) or directed against CaSR (siCaSR) for 48 h. CaSR inhibition is confirmed by an absence of immunostaining, and led to a decrease in NSE expression, from focal to almost diffuse.

To confirm the implication of CaSR in NE differentiation, transfection with siCaSR was performed in PC3 and 22RV1 cell lines. Unfortunately, the NCI-HH60 did not survive transfection. The inhibition of CaSR expression by siRNA was −62% and −51% in PC3 and 22RV1 cell lines, respectively (Figure 5A).

In siCaSR-transfected PC3 cells, the expression of neuronal markers NSE and βtubulinIII was reduced by 28% and 44%, respectively, and NE markers remained undetectable. CaSR inhibition in the 22RV1 cell line led to decrease the expression of NSE (−30%), synaptophysin (−31%) and chromogranin (−41%) (Figure 5A). All together, these results suggested that CaSR could be involved in the NE differentiation process.

To confirm these results, we analyzed protein expression of CaSR and NSE by immunohistochemistry (Figure 5B). The results confirm the efficiency of the siRNA directed against CaSR, with a strong decrease of CaSR expression in 22RV1. Interestingly, CaSR inhibition also led to a decrease of NSE staining.

## 3. Discussion

CaSR expression has been previously demonstrated by using immunoblotting in several PCa cell lines and has been shown to be increased in prostate cancer tissues compared to normal prostate [8,16]. A single report has investigated CaSR expression in human PCa by immunohistochemistry, with a positive staining at diagnosis associated with an increased risk of lethal disease [17].

In the present study, we observed in the epithelial compartment of the normal prostate a focal CaSR staining with less than 2% of positive cells. Normal glands with CaSR positive cells also showed a focal staining for chromogranin, in the same areas. In all groups, from normal tissue to NEPC, synaptophysin staining was observed in around half of chromogranin positive cases, suggesting that chromogranin is a better neuroendocrine marker than synaptophysin in the prostate. In NEPC, other studies, composed mainly of de novo cases, had in contrast reported a more frequent positivity for chromogranin than for synaptophysin [18,19]. This discrepancy could be explained by a different NE profile in post treatment versus de novo NEPC, most of our patients having been previously treated by hormonal deprivation. In conventional PCa with focal NE differentiation, the predominant chromogranin expression in NE cells when compared to synaptophysin has been previously described [20].

Colocalization of chromogranin and CaSR in the same tumor areas was also observed in CLC and MCRPC cases, and CaSR was expressed in all cases of NEPC. Double staining demonstrated that—particularly in NEPC and in MCRPC, where clusters of NE cells are found—most of CaSR positive cells in PCa have a neuroendocrine phenotype.

In human PCa tissues, we correlated CaSR expression with cancer cell proliferation and ERG expression that reflect the presence of the fusion gene TMPRSS2-ERG (observed in around half of PCa cases). We observed in the MCRPC group an inverse correlation between CaSR and both the proliferation and ERG expression. This association was also observed between chromogranin and ERG. In MCRPC, when present, NE cells were largely minority, with a median of 2% of cancer cells. Therefore, the decrease in proliferation we observed may concern non-NE neighboring cancer cells. Such an inhibitory effect of NE cells on the proliferation of adenocarcinoma cells has already been described in cell lines [21] and highlights the paracrine function of NE cells. Inversely, adenocarcinoma cells could also have an impact on the neuroendocrine component. In fact, it has been shown in a transgenic mouse model that ERG expression that reflects the presence of the TMPRSS2-ERG gene fusion, is able to decrease NE cell differentiation [22]. This report is perfectly in accordance with our present observation of decreased chromogranin and CaSR expression in ERG positive PCa. In both the CLC and NEPC groups, we found no significant association between CaSR staining, proliferation and ERG status. In CLC, the median percentage of CaSR positive cancer cells is less than 1%; it is therefore likely that such a tiny component could not able to influence the proliferation of the neighboring non-NE cancer cells. Inversely, in NEPC, all cases expressed CaSR, with a median of 90% positive cells. Therefore, it is understandable that, considering the high and rather homogeneous CaSR expression in NEPC, no association could be found between CaSR staining, the proliferation rate and ERG status (expressed in only 2 cases on 15).

CaSR staining in cancer cells increases with the stages of PCa progression, from pT2 to pT3 in CLC, then in MCRPC and NEPC. We also observed a similar increase in chromogranin expression between CLC and CRPC, as previously described [23,24,25]. It has been shown that the level of serum chromogranin A is associated with a bad prognosis in CRPC patients treated with either enzalutamide or abiraterone [26,27]. Treatment induced NEPC have a decreased overall survival compared to other CRPCs [28], but it remains uncertain whether focal neuroendocrine divergent differentiation in otherwise standard adenocarcinoma has a prognostic value. In CLC, after adjusting for usual prognostic factors such as ISUP group and pTNM stage, we found no significant correlation between disease free survival and either chromogranin or CaSR staining. In this group of patients, after adjustment, the only marker associated with biochemical recurrence was the proliferation marker Ki67, as previously shown [29]. On the other hand, in MCRPC, the CaSR status was significantly associated with decreased overall survival, while chromogranin staining was not. In six of our MCRPC specimens, CaSR is found to be expressed without chromogranin staining. Moreover, double immunostaining demonstrated that in some cells, CaSR can be expressed without chromogranin staining. These findings suggest that CaSR expression could occur earlier than other NED markers after castration and could be used for prognostic purpose.

It has been suggested that CaSR can act either as a tumor suppressor or as an oncogene, depending on the type of cancer. In colon carcinoma, CaSR is downregulated during tumorogenesis, leading to growth suppressive effects of high Ca^2+^ [30]. On the other hand, CaSR activation could facilitate bone metastasis in breast, kidney and prostate cancer [31,32,33]. CaSR could be targeted through NPS2143, a calcilytic drug with a good specificity and minor side effects [34], that has been shown to inhibit in vitro both proliferation and migration of the PCa cell line PC3 [35]. The link between a neuroendocrine phenotype, Ca^2+^ signaling and CaSR has already been suggested. In fact, NE differentiation in PCa cells has been associated with modifications of Ca^2+^ homeostasis [11], and CaSR has been shown to regulate hormone secretion in the enteroendocrine cells of the gastrointestinal tract [14]. The understanding of signal pathways involved in regulating NE differentiation in PCa is hampered by the lack of appropriate animal models or cell lines. NCI-H660, the only widely available NEPC cell line, is derived from a human NEPC, that expresses the PCa-specific TMPRSS2-ERG gene fusion [36]. This cell line is AR negative and expresses high levels of NE markers. However, its growth in vitro is slow—in the form of suspending cells—and transfection with plasmids or siRNA is not very effective, which limits its applications. We have therefore completed the experiments by using the PC3 PCa cell line that expresses neuronal markers, and the 22RV1 cell line that expresses high levels of both neuronal and NE markers.

We observed that CaSR is expressed in the three cell lines. Immunohistochemical staining shows that CaSR was not only localized to the plasma membrane but also in the intracellular compartment. The intracellular pool of receptor that localizes to the ER/Golgi is described as an intracellular reservoir, a mechanism thought to regulate membrane CaSR abundance in the presence of agonist [37]. As previously described, G-protein-coupled receptor (GPCR) are not confined to the plasma membrane and are capable of trafficking among a variety of subcellular compartments, initiating specific signaling pathways at different locations: nuclear, mitochondrial and the endosomal–Golgi membrane [38]. As observed in the present study, CaSR was also localized in the nuclear compartment in some cells and at a lower extend, but its biologic relevance in the nucleus remains to be explored. Activation of CaSR in PC3 cells led to induce a NE phenotype, with increased expression of NSE and synaptophysin. In addition, inhibition of CaSR by siRNA led to decrease the expression of neuronal markers in PC3 and of both neuronal and NE markers in 22RV1. Interestingly, the treatment of NCI-H660 and 22RV1 by the CasR inhibitor (NPS-2143) is able to decrease the expression of both neuronal and NE markers, suggesting that CaSR inhibition should reverse or limit the NE differentiation process. Taken together, these results suggest that CaSR could be a driver of NE differentiation in PCa cells. Several recent studies have proposed various mechanisms of NEPC development, including modifications of the tumor microenvironment with epigenetic effects on cancer cells, as well as reports of molecular drivers such as SRRM4, FOXA1 and ONECUT2 [39]. The connection of CaSR with the previously described molecular mechanisms of NE differentiation remains now to be determined. Since NE differentiation in PCa is mainly observed as a consequence of androgen targeted therapy, it will be necessary to understand the mechanisms initiating treatment induced CaSR expression in PCa cells.

In conclusion, our results demonstrate that CaSR is a marker of NE differentiation in PCa. CaSR expression is increased in aggressive disease states such as MCRPC and NEPC, is associated with decreased survival, and could therefore be targeted through specific antagonists.

## 4. Materials and Methods

### 4.1. Patients and Samples

Normal prostate tissues (*n* = 57) were obtained from patients treated by cystoprostatectomy for bladder carcinoma, without incidental prostate cancer. Written informed consents were obtained from patients following the requirements of the medical ethic committee of our institution (Comité de Protection des Personnes (CPP) de Tours—Région Centre Ouest I) (Ethic Code: DC-2014-2045) (years between 2003 and 2018). Clinically localized cancer samples (CLC) (*n* = 314), were obtained from patients treated by radical prostatectomy for localized PCa at Tours University Hospital and Institut Mutualiste Montsouris. CLC cases were composed of 157 tumors with negative surgical margins and biochemical relapse (defined as 2 consecutive increases in serum PSA 0.2 ng/ml or greater), matched with 157 tumors without recurrence. Each patient with biochemical relapse was matched with 1 patient with identical age groups, preoperative PSA, ISUP group and pathological stage, but who was free of recurrence after at least the same follow-up. This matching allows to have already taken into account the traditional predictive markers, in order to analyze the prognostic value of candidate markers. The median time to recurrence was 19 months (range 2–90) and the median follow-up in the group of patients without recurrence was 55 months (range 25–95).

Fifty-two cases of metastatic castration resistant prostate cancers (MCRPC) were selected from patients treated with androgen deprivation therapy (ADT). Hormonal relapse was defined as 2 consecutive rises in PSA, with serum testosterone under castration level (50 ng/dl). A total of 81 samples were analyzed, with 1 to 4 samples per patient. Tissues were collected either by transurethral resection, performed because of lower urinary tract symptoms associated with local tumor progression (*n* = 56) or by biopsy from a metastatic site (*n* = 15), including lymph nodes (*n* = 7), visceral (*n* = 7) and bone (*n* = 1) metastases. The median follow-up after tissue collection was 9.5 months (range 1–113). The median overall survival from tissue collection to death was 6.5 months (1–33 months). Cases with prominent NE cell differentiation were excluded from this group and classified as treatment induced NEPC.

Fifteen cases of neuroendocrine prostate cancer (NEPC) were selected from samples obtained by transuretral resection (*n* = 10), radical prostatectomy (*n* = 2) or prostate biopsies (*n* = 3). The diagnosis of NEPC was performed in case of prominent NE cell population, identified on morphologic criteria (small cells) and after immunostaining with either chromogranin or synaptophysin. Six cases were diagnosed de novo, in the absence of any prior treatment; 9 cases were subsequent to previous hormone deprivation. Six cases were pure NEPC; for 9 patients NEPC was mixed with conventional adenocarcinoma. All patients died; the median overall survival from tissue collection to death was 8 months (0.3–33 months).

The characteristics of patients and tissues are summarized in Table 2. Written informed consents were obtained from patients following the requirements of the medical ethic committee of our institution.

### 4.2. Immunohistochemistry on Tissue Micro-Array and Cell Lines

TMA Construction: TMAs were constructed using formalin-fixed paraffin-embedded tissue samples. Original slides stained with hematoxylin-eosin were reviewed using the 2009 TNM classification and the 2014 modified “Gleason” system. For each case, a minimum of 3 cores (0.6 diameter) were transferred from the selected areas to the recipient block, using a TMA workstation (Manual Tissue Arrayer MTA Booster, Alphelys, France). Serial 3 µm sections of the TMA blocks were used for immunohistochemistry. One section on ten was stained with hematoxylin-eosin to check that the cores adequately represented diagnostic areas.

Cell line pellets: cells were washed with a Phosphate Buffered Saline solution (PBS) (CS1PBS00-01, Eurobio, Les Ulis, France) and were harvested by trypsinization using TrypLE Express (12605-028, Gibco, Waltham, MA, USA). Centrifugation at 1500 rpm during 5 min was performed to get a cell pellet, which was subsequently fixed in formalin, embedded in paraffin and cut in 3 µm sections.

Immunohistochemistry: Slides were deparaffinized, rehydrated and heated in citrate buffer pH 6 for antigenic retrieval. After blocking for endogenous peroxidase with 3% hydrogen peroxide, the primary antibodies were incubated. The panel of primary antibodies included chromogranin (Zytomed, Berlin, Germany, MSG 062, 1/1, 30 min), synaptophysin (DakoCytomation, Glostrup, Denmark, clone DAK-SYNAP, 1/200, 30 min), CaSR (Thermofisher, Waltham, MA, USA, clone 5C10 ADD, 1/2000, 1 h), ERG (Roche Ventana, Oro Valley, AZ, USA, EPR 3864, 1/1, 30 min) and the proliferation marker Ki67 (DakoCytomation, Glostrup, Denmark, clone 39-9, 1/50, 30 min). Immunohistochemistry was performed with either the automated BenchMark XT slide stainer (Ventana Medical Systems Inc, Oro Valley, AZ, USA) using OptiView Detection Kit (Ventana Medical Systems Inc) or manually (for CaSR) using the streptavidin-biotin-peroxidase method with diaminobenzidine as the chromogen (Kit LSAB, Dakocytomation, Glostrup, Denmark). Slides were finally counterstained with hematoxylin. Negative controls were obtained after omission of the primary antibody or incubation with an irrelevant antibody. Positive control for CaSR staining was obtained by using sections of parathyroid tissue.

Double sequential immunostaining was performed using a double stain polymer kit (Zytomed plus 2-step, POLD2S). The first primary antibody (CaSR, mouse monoclonal antibody, Thermofisher, Waltham, MA, USA, clone 5C10 ADD) was applied at 1/2000 dilution and incubated for 1 h. After washing, the second primary antibody (chromogranin, rabbit monoclonal antibody, Abcam, Cambridge, UK, EP 1030Y) was incubated at 1/1000 dilution for 1 h. Horse radish peroxidase (HRP) polymer anti mouse was then applied for 30 min, followed by 10 min incubation with permanent HRP green kit (Zytomed, ZUC 070) as a substrate. Then alkaline phosphatase (AP) was incubated for 30 min, followed by permanent AP red chromogen (Zytomed, Berlin, Germany, ZUC 001). Contrasting chromogens were used to visualize the antibodies, green for CaSR and red for chromogranin. Scoring of antibody staining in TMA: The slides were analyzed by 2 observers in a blinded fashion. Chromogranin, CaSR and Ki67 were expressed as a percentage of total epithelial cells in case of normal tissues, or cancer cells in case of PCa. Synaptophysin and ERG were expressed as positive or negative. In case of inter-observer variability (different categories in the case of categorical data or variability more than 10% in the case of continuous data), which occurred in < 5% of cases, slides were rescored until a consensus was reached.

### 4.3. Cell Culture and Products

The PCa lines PC3 (CRL-1435), 22RV1 (CRL-2505) and NCI-H660 (CRL-5813) were purchased from American Type Culture Collection (ATTC-HTB-81). PC3 and 22RV1 were cultured in Roswell Park Memorial Institute medium (RPMI, BE12-702F, Lonza, Levallois-Perret, France) supplemented with 5% (PC3) or 10% (22RV1) fetal bovine serum (FBS) (CVF5VF00-01, Eurobio, Les Ulis, France) and 1% penicillin-streptomycin (PS). NCI-H660 were grown in RPMI 1640 Medium (ATCC modification, A10491-01) supplemented with: insulin (0.005 mg/mL); Transferrin (0.01 mg/mL); Sodium selenite (30 nM); Hydrocortisone (10 nM); β-estradiol (10 nM); L-glutamine (2 mM); 5% FBS and 1% PS. All cell lines were maintained in 37 °C humidified incubator with 5% CO_2_. The positive allosteric modulator of the CaSR, R-568 (SML2160), was purchased from Sigma-Aldrich (St-Quentin Fallavier, France). The CaSR inhibitor, calcilytic NPS-2143 hydrochloride (362610), was purchased from Tocris Bioscience (Bristol, United Kingdom).

### 4.4. RNA Extraction and Quantitative Real Time PCR

Total RNA was isolated with the Nucleopsin RNA kit (Macherey–Nagel, Hoerdt, France) according to the manufacturer’s protocol. RNA concentration and purity were determined by measuring the absorbance at 230, 260 and 280 nm with spectrophotometer EpochTM (Biotek Instruments, Inc, Colmar, France). To obtain cDNA at 50 ng/µL, RNAs were reverse transcribed with RT kit (PrimeScriptTM RT Reagent, Perfect Real Time, Takara, Saint Quentin Yvelines, France). The reaction progressed in a LightCycler 480 (Roche Applied Science, Meylan, France) at 37 °C during 17 min followed by 85 °C for 5 min. For reactions, 5 µL of SYBR Green mix (RR420L, Takara, Saint Quentin Yvelines, France) was mixed with 1 µL of specific primers (0.5 µL of each primer: forward and reverse) and 1 µL of cDNA at 50 ng/µL. The quantitative PCR analysis was done using CFX ConnectTM BIORAD and analyzed with Biorad CFX Maestro software. Relative levels of mRNA were calculated according to the ΔΔCT method, relative to the housekeeping gene HPRT (hypoxanthine phosphoribosyltransferase) and TBP (TATA-binding protein). Primers used for quantitative real time PCR are available in Table S1.

### 4.5. Transfection Assays

Cells were plated in culture dishes in culture medium at a density of 170 000 cells/well, 24 h before transfection. Cells were incubated with a mix of siRNA and lipofectamineTM RNAiMAX (10514953, Fisher Scientific TM, Illkirch, France) in medium for 24 h. On the next day, medium was refreshed. SiRNA used in this study are available in Table S2. Experiments were performed 48 h after transfection. PCR and immunohistochemical analysis of cells were performed 48 h after transfection.

### 4.6. Statistical Analysis

Statistical analyses were carried out with StatView, version 5.0, software (Abacus Concepts, Berkeley, CA, USA). Comparison between groups was performed using the χ2 test for categorical data and nonparametric Mann–Whitney U test or Kruskall–Wallis test for continuous data. The relationship between 2 continuous variables was evaluated using the nonparametric Spearman rank correlation test. Recurrence after treatment was analyzed by Kaplan-Meier method, and the curves were compared using the log-rank test. *p*-values < 0.05 were considered significant.

## 5. Conclusions

The present study demonstrate that CaSR is a marker of NE differentiation in PCa. CaSR expression is increased in aggressive disease states such as MCRPC and NEPC, is associated with decreased survival, and could therefore be targeted through specific antagonists.

## Figures and Tables

**Table 1:** Markers expression in prostate tissue samples.

	NL (*n* = 57)	CLC (*n* = 314)	MCRPC (*n* = 81)	NEPC (*n* = 15)
**CaSR**				
**− (*n*)**	33	231	61	0
**+ (*n*)**	24	83	20	15
**% (median, range)**	1 (1–2)	1 (1–8)	2 (1–15)	90 (40–100)
**Chromogranin**				
**− (*n*)**	34	227	67	0
**+ (*n*)**	23	87	14	15
**% (median, range)**	1 (1–2)	1 (1–9)	2 (1–7)	80 (10–100)
**Synaptophysin**				
**− (*n*)**	45	269	72	9
**+ (*n*)**	12	42	9	6
**Ki67**	NA			
**% (median, range)**		2 (0–12)	6 (0–70)	70 (8–90)
**ERG**	NA	(274 cases available)		
**− (*n*)**		114	50	13
**+ (*n*)**		60	31	2

CaSR: calcium-sensing receptor; NL: normal prostate tissue; CLC: hormone naive clinically-localized cancer; MCRPC: metastatic castration resistant prostate cancer. NEPC: neuroendocrine prostate cancer; NA: not applicable.

**Table 2:** Patients and tissues characteristics.

Groups	NL (*n* = 57)	CLC (*n* = 314)	MCRPC (*n* = 52)	NEPC (*n* = 15)
**Age (year), Median (Range)**	64 (48–80)	63 (46–74)	69 (48–91)	77 (61–90)
**PSA (ng/ml)**	-	9 (1.5–22)	31.6 (3.2–2000)	5.8 (0.03–12.8)
**pTNM**	NA		NA	NA
pT2		204		
pT3		110		
**ISUP Group**	NA		NA	NA
1		77		
2		89		
3		128		
4–5		20		

NL: normal prostate tissue; CLC: hormone-naive clinically-localized cancer; MCRPC: metastatic castration resistant prostate cancer; y: years; NEPC: neuroendocrine prostate cancer; NA: not applicable.

## Supplementary Materials

The following are available online at https://www.mdpi.com/2072-6694/12/4/860/s1, Table S1: Primers used for quantitative real-time PCR; Table S2: siRNA sequences used for transfection assays.
