# Supplementary figures and images for: The Calcium-Sensing Receptor is A Marker and Potential Driver of Neuroendocrine Differentiation in Prostate Cancer
Source: Cancers (Basel). 2020 Apr 2;12(4):860. doi: 10.3390/cancers12040860 (PMC7226072; doi:10.3390/cancers12040860)

**Supplementary Materials**

**
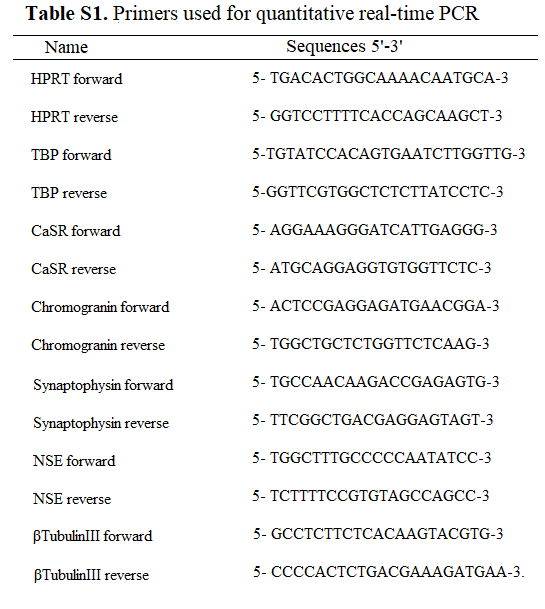
**

**
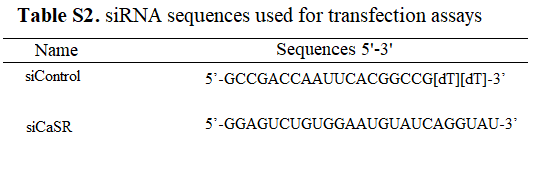
**

Supplement: Supplementary file 1 [file cancers-12-00860-s001.zip › cancers-754951-supplementary.docx]
